# Intradermal vaccination of live attenuated influenza vaccine protects mice against homologous and heterologous influenza challenges

**DOI:** 10.1038/s41541-021-00359-8

**Published:** 2021-08-04

**Authors:** Andrew Chak-Yiu Lee, Anna Jinxia Zhang, Can Li, Yanxia Chen, Feifei Liu, Yan Zhao, Hin Chu, Carol Ho-Yan Fong, Pui Wang, Siu-Ying Lau, Kelvin Kai-Wang To, Honglin Chen, Kwok-Yung Yuen

**Affiliations:** 1 Department of Microbiology, Li Ka Shing Faculty of Medicine, The University of Hong Kong, Hong Kong, China; 2 Research Centre of Infection and Immunology, The University of Hong Kong, Hong Kong, China; 3 State Key Laboratory of Emerging Infectious Diseases, The University of Hong Kong, Hong Kong, China

**Keywords:** Vaccines, Live attenuated vaccines, Influenza virus

## Abstract

We previously developed a temperature-sensitive, and NS1 gene deleted live attenuated influenza vaccine (DelNS1-LAIV) and demonstrated its potent protective efficacy in intranasally vaccinated mice. Here we investigated whether intradermal (i.d.) vaccination induces protective immunity. Our results showed that DelNS1-LAIV intradermal vaccination conferred effective and long-lasting protection against lethal virus challenge in mice. A single intradermal injection of DelNS1-LAIV conferred 100% survival with no weight loss in mice after A(H1N1)09 influenza virus (H1N1/415742Md) challenge. DelNS1-LAIV injection resulted in a significant reduction of lung viral load and reduced airway epithelial cell death and lung inflammatory cytokine responses at day 2 and 4 post challenge. Full protections of mice lasted for 6 months after immunization. In vitro infection of DelNS1-LAIV in monocyte-derived dendritic cells (MoDCs) demonstrated activation of antigen-presenting cells at 33 °C, together with the results of abortive replication of DelNS1-LAIV in skin tissue and strong upregulation of inflammatory cytokines/chemokines expression, our results suggested the strong immunogenicity of this vaccine. Further, we demonstrate that the underlying protection mechanism induced by intradermal DelNS1-LAIV is mainly attributed to antibody responses. Together, this study opens up an alternative route for the administration of LAIV, which may benefit individuals not suitable for intranasal LAIV immunization.

## Introduction

The World Health Organization estimated that seasonal influenza causes about one billion infections globally each year, with 0.3–0.7 million deaths^1^. Despite the availability of several classes of antiviral drugs such as neuraminidase inhibitors and polymerase inhibitors, vaccination is still the most effective option for controlling influenza epidemic and pandemic. Currently, inactivated influenza vaccines and live attenuated influenza vaccines (LAIV) are administrated mainly through intramuscular injection and intranasal spray, respectively^2^. Both approaches are shown to be immunogenic. LAIV has been licensed for clinical use since 2003. Besides avoiding an unpleasant injection, intranasal LAIV immunization mimics the process of a natural infection. Attenuated vaccine virus infects and replicates in the upper respiratory epithelial cells to elicit systemic and local antiviral immune responses^3,4^. In some individuals, transient local side effects were observed up to 2–3 days after intranasal (i.n.) vaccination^3,5^ but were generally well tolerated. However, LAIV is not recommended for children under 2 years old, people above 49 years old, pregnant women, and people with asthma or chronic obstructive lung diseases or immunocompromised conditions because these temperature-sensitive vaccine viruses with intact viral NS1 gene may replicate to higher titer in the respiratory tract to cause severe side effects^2^. Another potential risk is that vaccine virus may reassort with wild-type influenza viruses if simultaneous natural infection and immunization occur, although the progeny virus is unlikely to be transmissible^6^.

Besides the nasal respiratory mucosa, the skin is another immune organ which harbors lots of innate immune cells including Langerhans cells, dermal dendritic cells, and residential B cells^7^. Vaccines other than influenza vaccine delivered to skin have been proven to induce potent protective immunity^8^. Inactivated influenza vaccine through intradermal route is only regaining more interests in recent years owing to the development of new technologies and new devices for easier, more reliable intradermal delivery^9,10^. Our previous clinical trials for intradermal inactivated seasonal influenza vaccine with topical imiquimod cream demonstrated rapid, strong, and longer-lasting antibody responses^11,12^. Intradermal vaccination is antigen dose-sparing. Compared with other administration routes, i.d. injection could potentially reduce the cost per injection to about 60%^13,14^. This is of particular significance in vaccine resource-poor settings, especially during the start of a pandemic when global vaccine production capacity limits the supply^15^.

Live attenuated influenza vaccine DelNS1-LAIV is highly attenuated in human cell culture and non-pathogenic in mice because the virulence factor NS1 protein was deleted from the vaccine virus^16^. We reported previously that i.n. vaccination with DelNS1-LAIV provided full protection against homologous virus A(H1N1)pdm09 and heterologous H7N9 and H5N1 influenza challenges in mice^16^. DelNS1-LAIV is cold-adapted with limited replication at 33 °C which is similar to the temperature of human and mouse skin^17,18^. In this study, we evaluate whether i.d. DelNS1-LAIV vaccine can induce durable protective immune responses with homologous and heterologous protection. The findings may broaden the application of LAIV.

## Results

### Intradermal DelNS1-LAIV vaccination fully protected mice against homologous H1N1/415742Md challenge

**Fig. 1:**
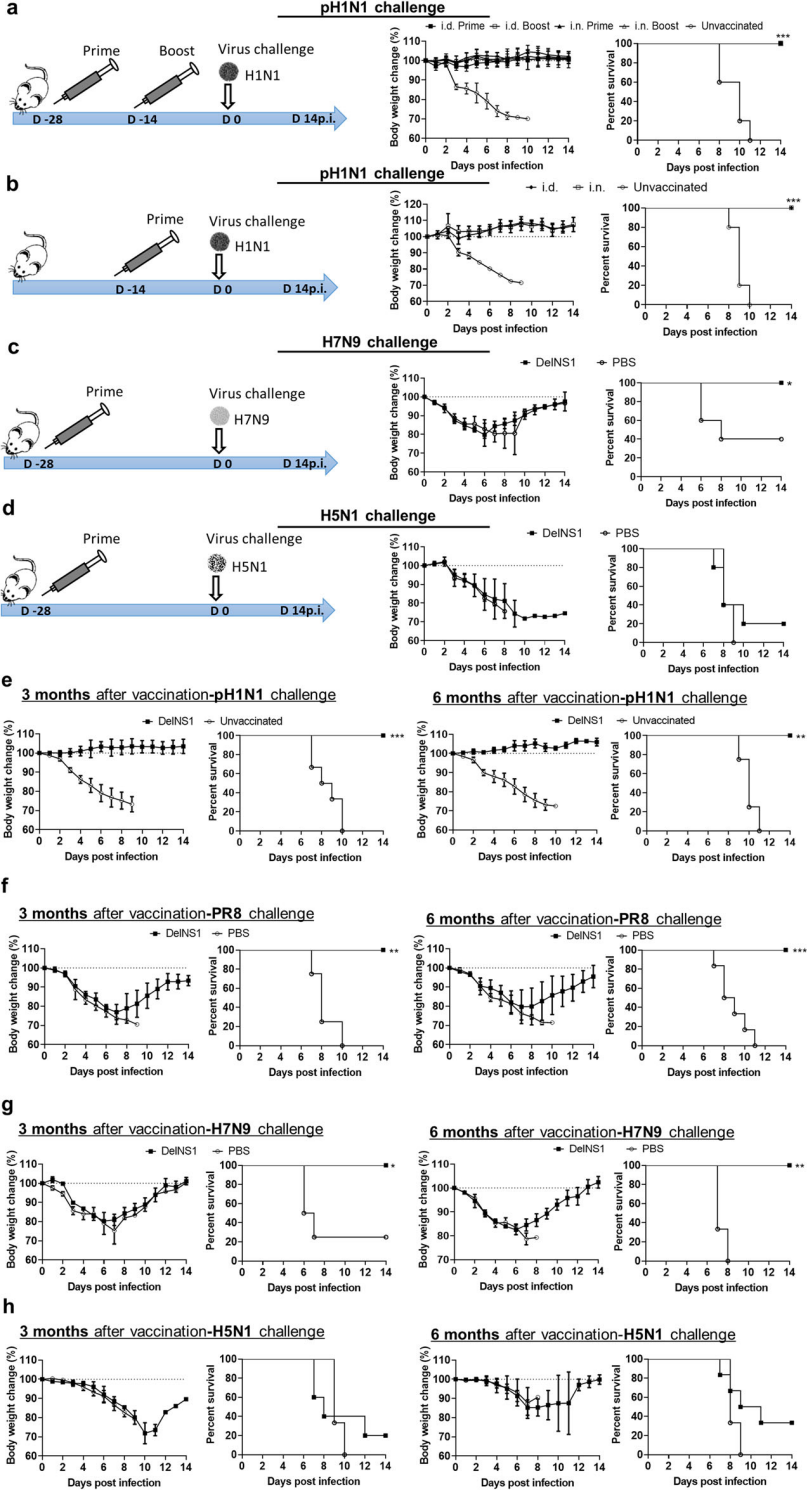
Intradermal vaccination of DelNS1-LAIV protected mice against homologues and heterologous virus challenge. **a** Schema for vaccination, body weight change, and survival rate for mice challenged by homologous virus A/Hong Kong/415742Md (pH1N1) 28 days after vaccination. **b** Vaccination schema, body weight change, and survival rate for mice challenged by A/Hong Kong/415742Md at 14 days after one dose vaccination. **c** A/Anhui/1/2013m (H7N9) challenged at 28 days after one dose vaccination. **d** A/VNM/1194/2004 (H5N1) virus challenged at 28 days after one dose vaccination. *n* = 4–5 mice per group. Error bars indicate standard deviation. ^*^p < 0.05; ^**^p < 0.01; ^***^p < 0.001 when compared with PBS or unvaccinated group by log-rank test. **e**–**h** Longevity of vaccination induced protection in mice. Body weight and survival for groups of mice challenged by: **e** A/Hong Kong/415742Md (pH1N1) virus, **f** A/Puerto Rico/8/1934 (PR8), **g** A/Anhui/1/2013m (H7N9), or **h** A/VNM/1194/2004 (H5N1) virus at 3 months or 6 months after one dose vaccination. *n* = 4–6 mice per group. Error bars indicate standard deviation. ^*^p < 0.05; ^**^p < 0.01; ^***^p < 0.001 when compared with the survival of PBS group by log-rank test.

To test whether LAIV is effective via intradermal route, 10^6^ plaque forming units (PFU) of DelNS1-LAIV was i.d. injected to multiple groups of mice. One of the groups was boosted with second injection at 14 days after the prime vaccination. Unvaccinated control mice were injected with the same volume of PBS. The mice were intranasally challenged with 10x LD_50_ of H1N1/415742Md at 28 days after primary vaccination. Both single dose and two doses of i.d. vaccination induced good protection with no weight loss and 100% survival after virus challenge (Fig. 1a). Comparing i.d. vaccinated mice with i.n. vaccinated mice, there was no difference in body weight loss or survival rate (Fig. 1a), which suggested LAIV i.d. vaccination offered the same protective efficacy as i.n. vaccination. Remarkably, a single dose of i.d. vaccination fully protected mice against virus challenge with 100% survival and no weight loss (Fig. 1b).

To test the broadness of i.d. vaccination-induced immunity, H7N9 (A/Anhui/1/2013m) or H5N1 (A/VNM/1194/2004) challenges were performed at 28 days after a single dose of i.d. vaccination. H7N9 challenge caused sharp weight loss and 60% death in PBS control mice, while the vaccinated mice were 3 days quicker in body weight recovery and had 100% survival (Fig. 1c). However, i.d. DelNS1-LAIV only rescued the survival rate to 20% among the H5N1-challenged mice, versus 100% mortality in the PBS group (Fig. 1d).

We then studied the longevity of i.d. DelNS1-LAIV-induced immunity by challenging the vaccinated mice at 3 or 6 months after vaccination. Firstly, homologues virus H1N1/415742Md challenge did not cause body weight loss, nor lethality 3 or 6 months after vaccination (Fig. 1e), suggesting the protective immunity lasted at least for 6 months; Secondly, all vaccinated mice survived against an antigenically different H1N1 strain (PR8) challenge and regaining body weight starting day 7 post challenge (7dpi) (Fig. 1f). All vaccinated mice survived after H7N9 challenge though with a similar degree of weight loss comparing to the PBS control mice (Fig. 1g). The immunized mice challenged by H5N1 at 3 or 6 months had 30% and 20% survival, respectively, with a similar degree of weight loss comparing to the PBS controls (Fig. 1h).

### DelNS1-LAIV intradermal vaccination controls virus replication and reduces histopathological damages in the virus-challenged lung

**Fig. 2:**
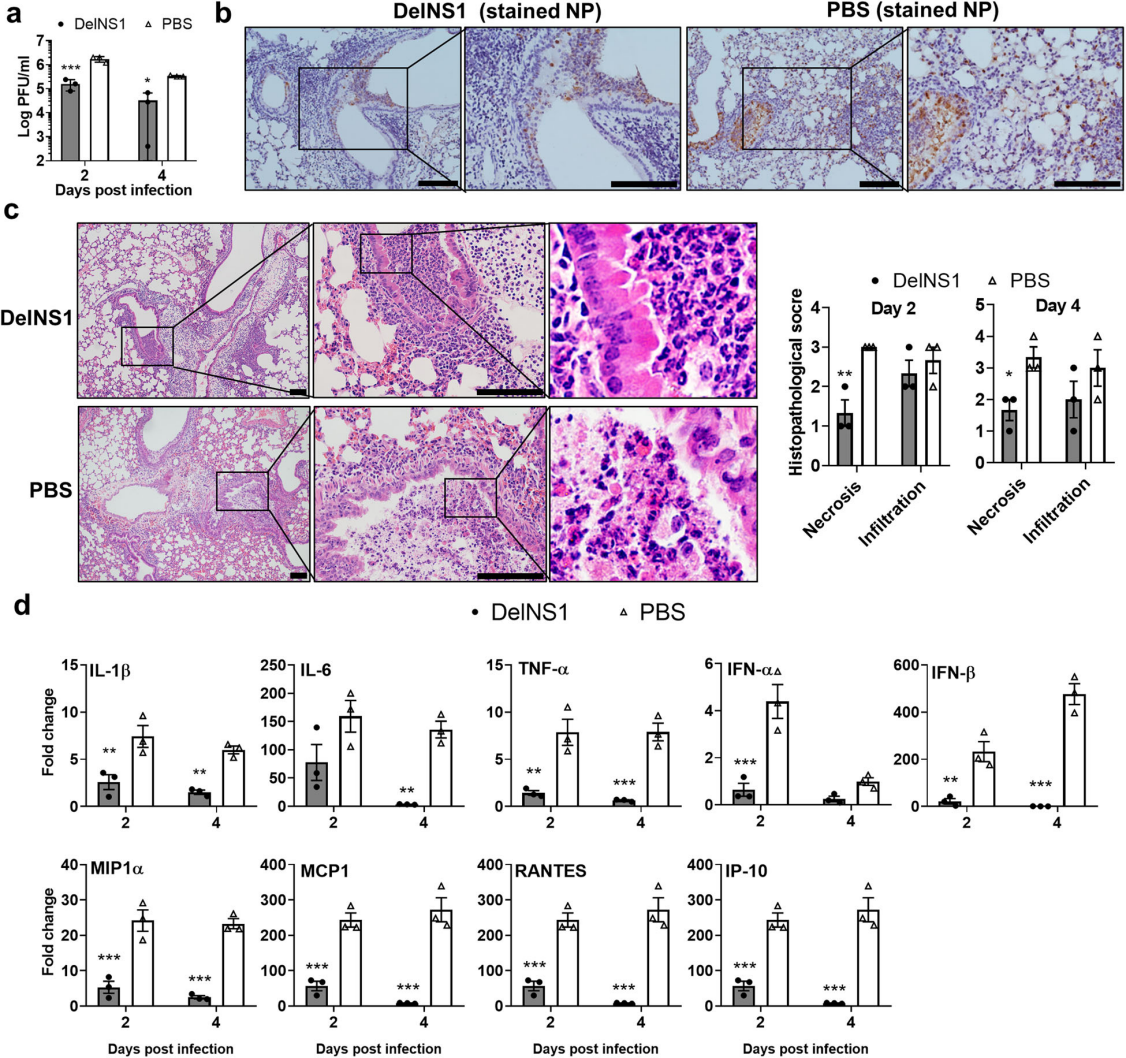
Viral load and lung histology of vaccinated mouse challenged with A/Hong Kong/415742Md (pH1N1). The mice were challenged with 10x LD_50_ of A/Hong Kong/415742Md 14 days after one dose i.d. vaccination. Lung samples were analyzed at 2 and 4 days post infection (dpi). **a** Lung infectious viral titer determined by plaque forming assay on MDCK cells. *n* = 3 mice per group. **b** Representative images of influenza nucleoprotein (NP) expression in the lung at 2 dpi stained by immunohistochemistry. Scale bars = 100 µm. **c** Representative images of hematoxylin and eosin-stained lung sections at 2 dpi. Scale bars = 100 µm. The chart on the right was histology scores, *n* = 3 for each group. **d** Real-time RT-PCR determined the relative expression of cytokines and chemokines in lung homogenate. Fold changes of the gene expression were relative to the uninfected mouse lung samples. *n* = 3 mice per group. Error bars indicate standard error of mean. ^*^p < 0.05; ^**^p < 0.01; ^***^p < 0.001 when compared with PBS group by two-way ANOVA.

The vaccinated mice had significantly reduced infectious virus titer in the lung tissue at 2 and 4 dpi comparing to the control mice (Fig. 2a). Vaccinated mouse lung sections also showed much less viral nucleoprotein (NP)-expressing cells. The NP-positive cells were mainly confined to the larger bronchial/bronchiolar epithelium, rarely affecting alveolar epithelium (Fig. 2b). In contrast, in the unvaccinated mouse lung sections, NP-positive cells were more extensively distributed in the airway epithelium and more frequently detected in alveolar epithelial cells (Fig. 2b), indicating that the dissemination of virus to distal structure of lungs was controlled in the vaccinated mice. Further, vaccinated mouse lung sections showed no severe pathological damages, significantly less bronchial and alveolar epithelial cell death, and less destruction of airway structure comparing to the PBS control (Fig. 2c), resulting in a significantly improved epithelium necrosis score (Fig. 2c). Furthermore, the expression levels of lung inflammatory cytokine/chemokine were significantly lower in the vaccinated mice than that in the unvaccinated mice (Fig. 2d).

### DelNS1-LAIV vaccine virus infection and activation of human monocyte-derived dendritic cells (MoDCs) at 33 °C

**Fig. 3:**
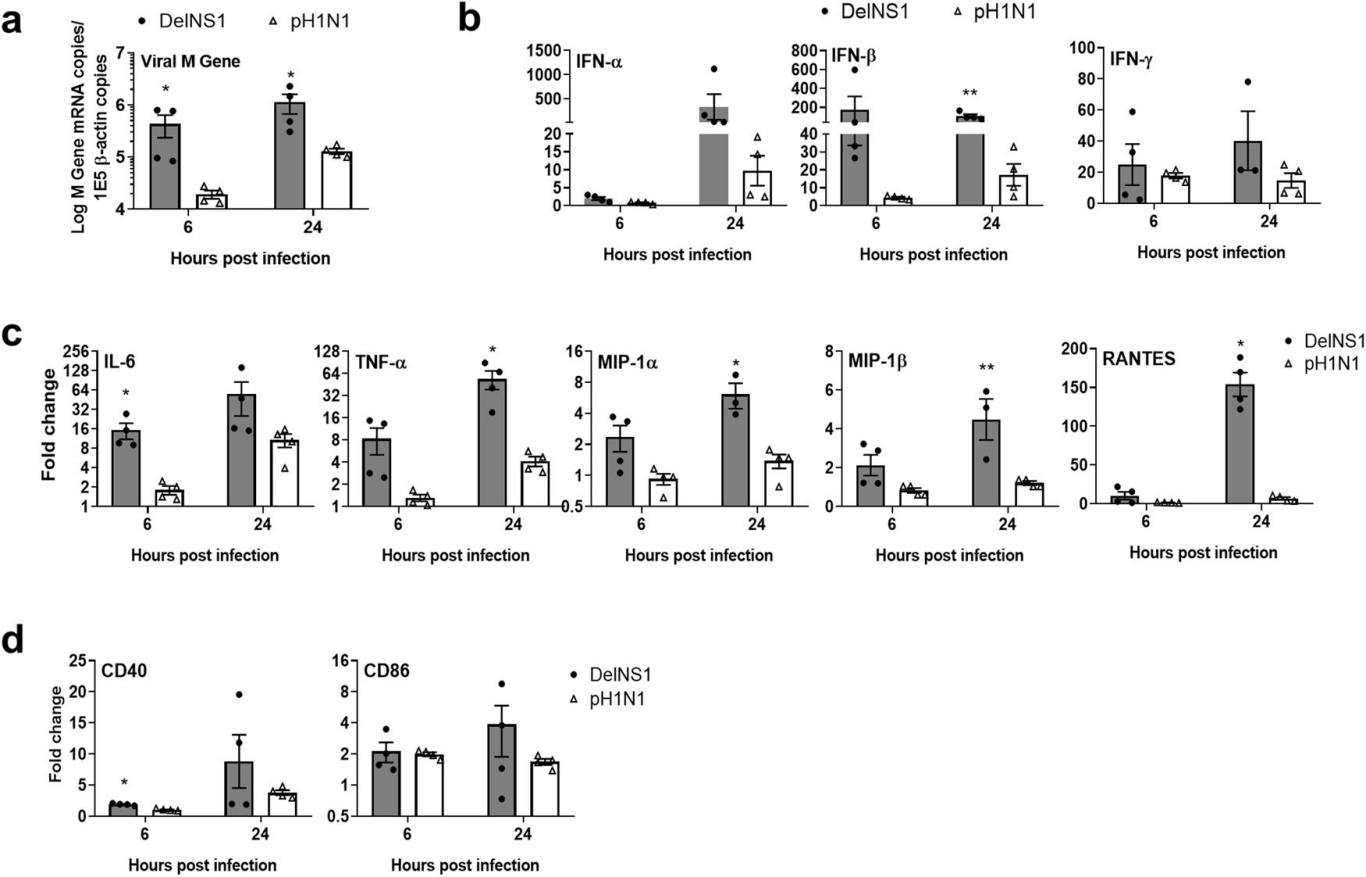
DelNS1-LAIV virus strain infection and activation of monocyte-derived dendritic cells (MoDCs) in vitro. Human monocyte-derived dendritic cells (MoDCs) were infected with 2 MOI of DelNS1-LAIV or wild-type A/Hong Kong/415742/2009 (pH1N1) at 33 °C. Cell lysates were collected 6 and 24 hours post infection (hpi). **a** Viral M gene mRNA copies in MoDCs determined by real-time RT-PCR. **b** Relative expression levels for interferon-α, -β, -γ; **c** Pro-inflammatory cytokines/chemokines. **d** Dendritic cell co-stimulatory molecules expression were also determined by real-time PCR. Fold changes of the genes expression were relative to the mock-infected MoDCs. *n* = 4 per group. Error bars indicate standard error of mean. ^*^p < 0.05; ^**^p < 0.01; ^***^p < 0.001 when compared with pH1N1 by two-way ANOVA.

DelNS1-LAIV virus is adapted to cold temperature and replicates at 33 °C^16^. However, the responses of immune cells to DelNS1-LAIV virus are unknown. To evaluate this, we inoculated human MoDCs^19^ with DelNS1-LAIV (MOI = 2), our results showed that DelNS1-LAIV replicated to a higher level than wild-type virus A/Hong Kong/415742/2009 at 33 °C (Fig. 3a). However, viral M gene mRNA copies increased only by less than half-log from 6 to 24 hours post infection (hpi) (Fig. 3a), indicating DelNS1-LAIV directly infected MoDCs but replicated to a limited degree. MoDCs were activated by DelNS1-LAIV infection and expressed significantly higher levels of IFN-α, IFN-β, and IFN-γ (Fig. 3b). Similarly, inflammatory cytokines including IL-6 and TNF-α, and chemokines including MIP-1α, MIP-1β, and RANTES were significantly upregulated at 6 hpi and further increased at 24 hpi in MoDCs (Fig. 3c). In addition, the expression of co-stimulatory molecules CD40 and CD86 on MoDCs increased at 24 hpi (Fig. 3d), which also indicated that DelNS1-LAIV could rapidly and effectively activate dendritic cells at 33 °C.

### DelNS1-LAIV induced strong innate immune responses in skin tissue and draining lymph nodes

**Fig. 4:**
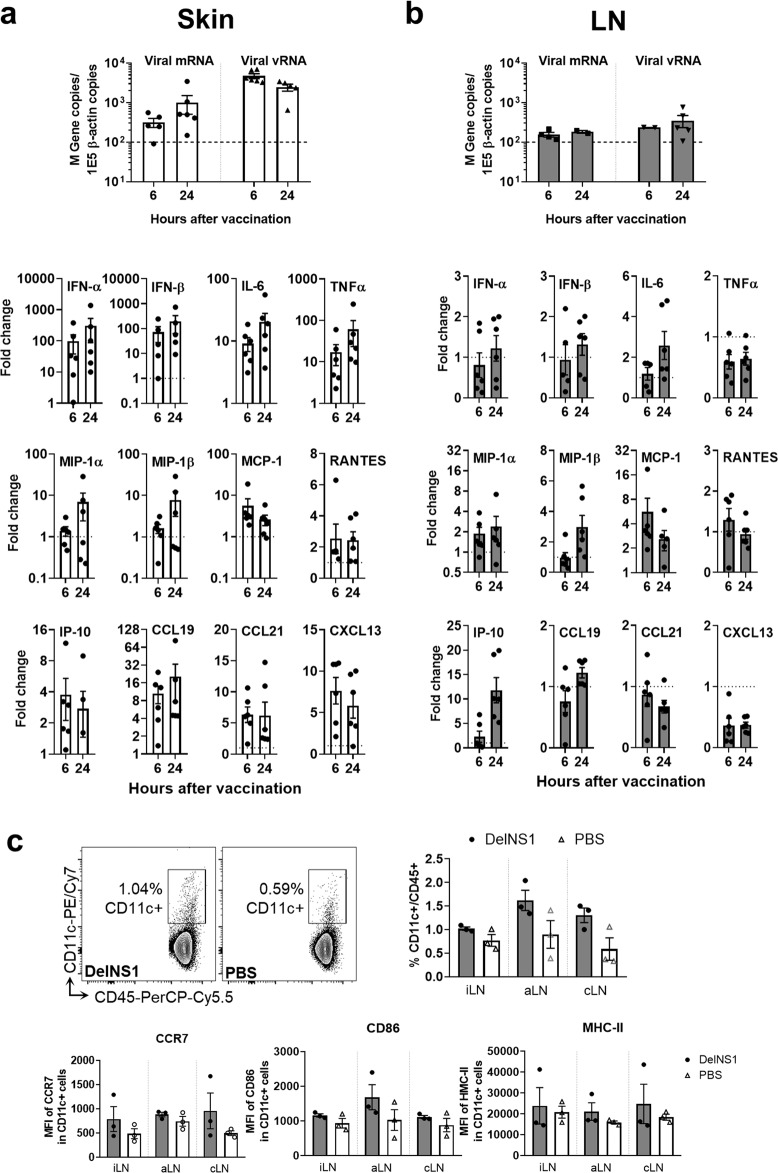
DelNS1-LAIV induced local innate immune responses in skin and skin-draining lymph nodes. Mouse skin tissue of the i.d. injection sites and skin-draining lymph nodes were harvested 6 and 24 h after DelNS1-LAIV or PBS injection. **a** Real-time RT-PCR determined the expression of viral vRNA, viral mRNA, and cytokines/chemokines in skin tissues. **b** The expression of viral vRNA, viral mRNA, and cytokines/chemokines in draining lymph nodes (pooled samples of inguinal lymph nodes, axillary lymph nodes, and superficial cervical lymph nodes). Dashed lines in the viral load chart indicated the detection limit of 100 copies of viral gene. The relative expression levels of cytokines/chemokines were relative to the PBS control mice, which were indicated by the dotted line in the charts. *n* = 6 mice per group. **c** Different sets of the inguinal (iLN), axillary (aLN), and superficial cervical (cLN) lymph nodes were harvested for dendritic cell analysis at 6 h after vaccine injection. The percentage of CD11c^+^ dendritic cells (DC) and mean florescence intensity of the expression levels for DC activation markers CCR7, CD86, and MHC-II were analyzed by flow cytometry. *n* = 4 per group. Error bars indicate standard error of mean.

Next, we analyzed the skin tissues and showed that influenza M gene mRNA increased from 6 to 24 h after i.d. injection, while M gene vRNA copies was higher at 6 h comparing to that at 24 h after vaccination (Fig. 4a, upper), suggesting abortive viral replication. The expression of cytokines/chemokines including IFN-α/β, IL-6, TNF-α, and MIP-1α/β in skin tissue (Fig. 4a, lower) significantly increased at 6 h after injection, and continued to increase at 24 h. MCP-1 expression was higher at 6 h than 24 h after injection, while RANTES, IP-10, and CCL19, CCL21, and CXCL13 were upregulated at similar levels at 6 or 24 h after injection. The upregulated expression of these cytokines and chemokines are consistent with reported molecular events induced by i.d. vaccination^20^, and suggested that a broad spectrum of innate immune responses at the injection site were elicited by i.d. vaccination. However, no visible or histological skin tissue damages were observed despite the upregulation of the inflammatory cytokines/chemokines (Supplementary Fig. 1).

Skin-draining lymph nodes including inguinal, axillary, and superficial cervical lymph nodes were analyzed. The level of viral M gene mRNA and vRNA in lymph nodes were just above our qRT-PCR detection limit, with no increase from 6 to 24 h after i.d. DelNS1-LAIV injection (Fig. 4b, upper), indicating a limited amount of live vaccine virus, if any, could reach the lymph nodes. Inflammatory cytokine/chemokine expression profiling showed mild upregulation of IL-6, MIP1α/β, MCP-1, and IP-10 at 24 h after i.d. vaccination. The expression levels of IFN-α/β, TNF-α, RANTES, CCL19, CCL21, and CXCL13 were just above the baseline levels (Fig. 4b, lower). The number of CD11c^+^ dendritic cells were slightly increased in axillary and superficial cervical lymph nodes at 6 h after i.d. vaccination, which had a higher expression levels of the cell migration marker CCR7. At the same time, the expression levels of co-stimulatory molecule CD86 and MHC-II molecule also increased in the CD11c^+^ dendritic cells (Fig. 4c). However, all of these changes did not reach statistical significance.

### Intradermal vaccination of DelNS1-LAIV induced adaptive antibody responses

**Fig. 5:**
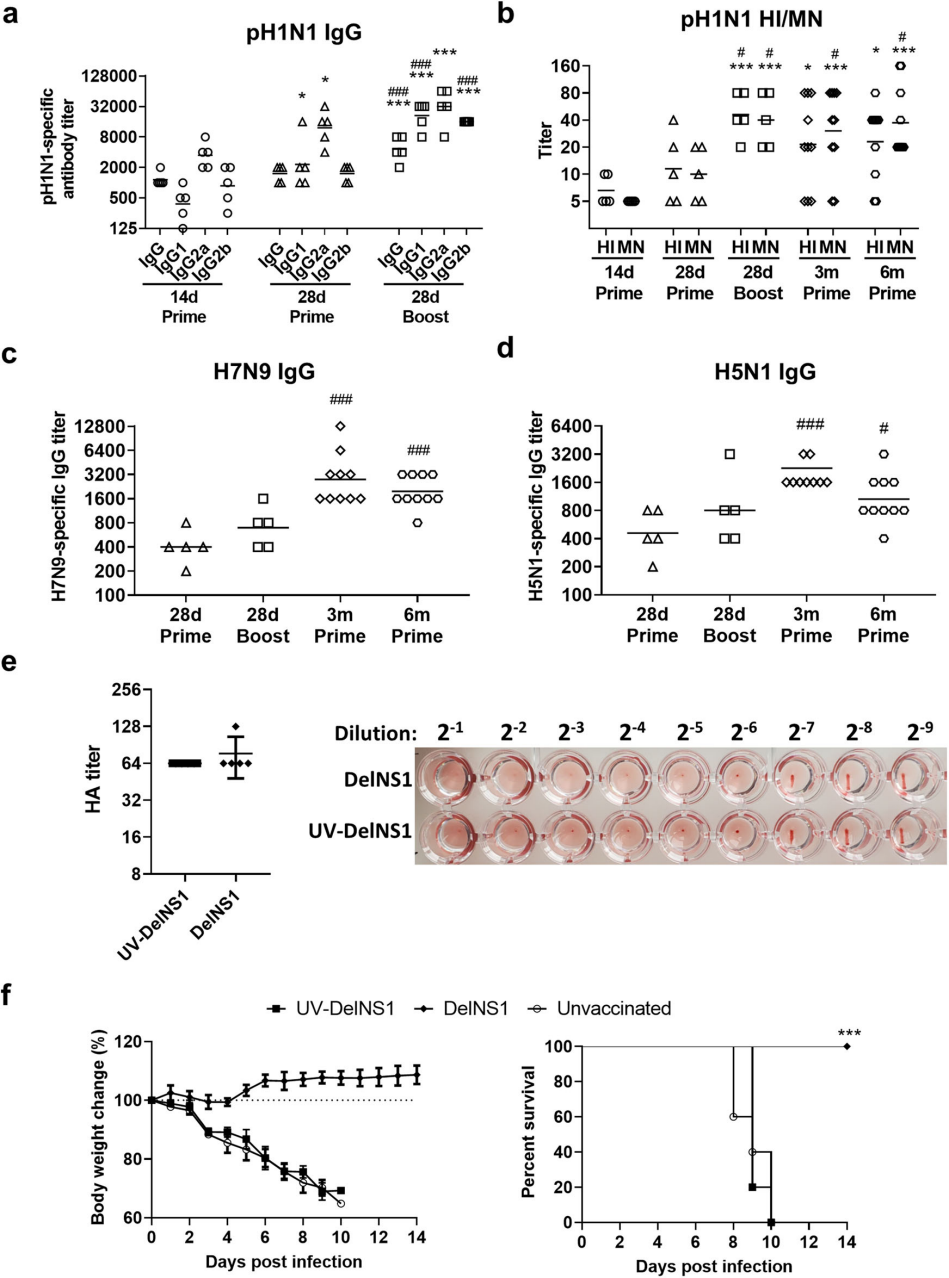
DelNS1-LAIV induced antibody response in mouse. Mouse serum samples were collected at 14 days, 28 days, 3 months, and 6 months after the first vaccination. **a** Geometric mean titer (GMT) of pandemic A/Hong Kong/415742 /2009 (pH1N1) virus-specific IgG and IgG subtypes in mouse serum detected by ELISA using inactivated pH1N1-coated plate. **b** GMT of hemagglutination inhibition (HI) and microneutralizing (MN) antibody titer against pH1N1 virus. GMT of serum virus-specific IgG antibody against H7N9 (**c**) or H5N1(**d**) virus determined by ELISA using inactivated H7N9 or H5N1 virus-coated plate, respectively. **a**–**d** *n* = 5 or 10 per group. ^*^p < 0.05; ^**^p < 0.01; ^***^p < 0.001 when compared with day 14 prime data; ^#^*p* < 0.05; ^###^*p* < 0.001 when compared with day 28 prime data using Student’s *t*-test on log transformed titers. **e** HA titer and image of HA assay showed UV inactivated DelNS1 virus (10^6^ PFU) did not change the HA titer (*n* = 5 repeated experiments). **f** Body weight changes and survival of mice. Groups of mice were intradermally vaccinated with same dose of UV-inactivated or live DelNS1-LAIV (10^6^ PFU) for 14 days and challenged with 10x LD_50_ of Hong Kong/415742Md virus. Body weight and survival rate of mice after challenge for 14 days. *n* = 5 mice per group. Error bars indicate standard deviation. ^***^p < 0.001 when compared with survival of UV-DelNS1 group by log-rank test.

Influenza virus-specific IgG antibody was detected in the serum by enzyme-linked immunosorbent assay (ELISA) at 14 days after vaccination, which were mainly IgG2a, followed by IgG2b and IgG1 isotypes. IgG2a and IgG1 further increased at 28 days after vaccination (Fig. 5a). The second dose of vaccination significantly boosted the concentration of total IgG, IgG2a, IgG2b, and IgG1 (Fig. 5a). Hemagglutination inhibition (HI) antibody titer against homologous virus was detected at 28 days after one dose of vaccination, and significantly increased after the second vaccination (Fig. 5b). Virus neutralizing titer was similarly detected at 28 days after vaccination, which was significantly increased after the second vaccination (Fig. 5b). At 3 and 6 months after the one dose vaccination, similar titers of serum HI and MN antibody were detected indicating the presence of long-lasting antibody secreting cells (Fig. 5b). Serum IgG against H7N9 (Fig. 5c, left) or H5N1 (Fig. 5c, right) were detected by ELISA at 28 days after vaccination, and further increased at 3 months and maintained at 6 months post vaccination.

Together, our findings suggested that live attenuated influenza virus given via intradermal route effectively induced protective humoral immunity. To confirm its relation with the higher immunogenicity of live vaccine, a same dose of UV-inactivated^21^ DelNS1 virus was i.d. injected to BALB/c mice and challenged with H1N1/415742Md 14 days after vaccination. Hemagglutination test showed no loss of HA titer after UV-inactivation (Fig. 5e). However, body weight change and survival data from the challenged mice showed that one dose (equivalent to 10^6^ PFU) of inactivated vaccine did not confer protection against lethal virus challenge (Fig. 5f).

### Intradermal vaccination of DelNS1-LAIV induced viral-specific T cell responses in challenged mice

**Fig. 6:**
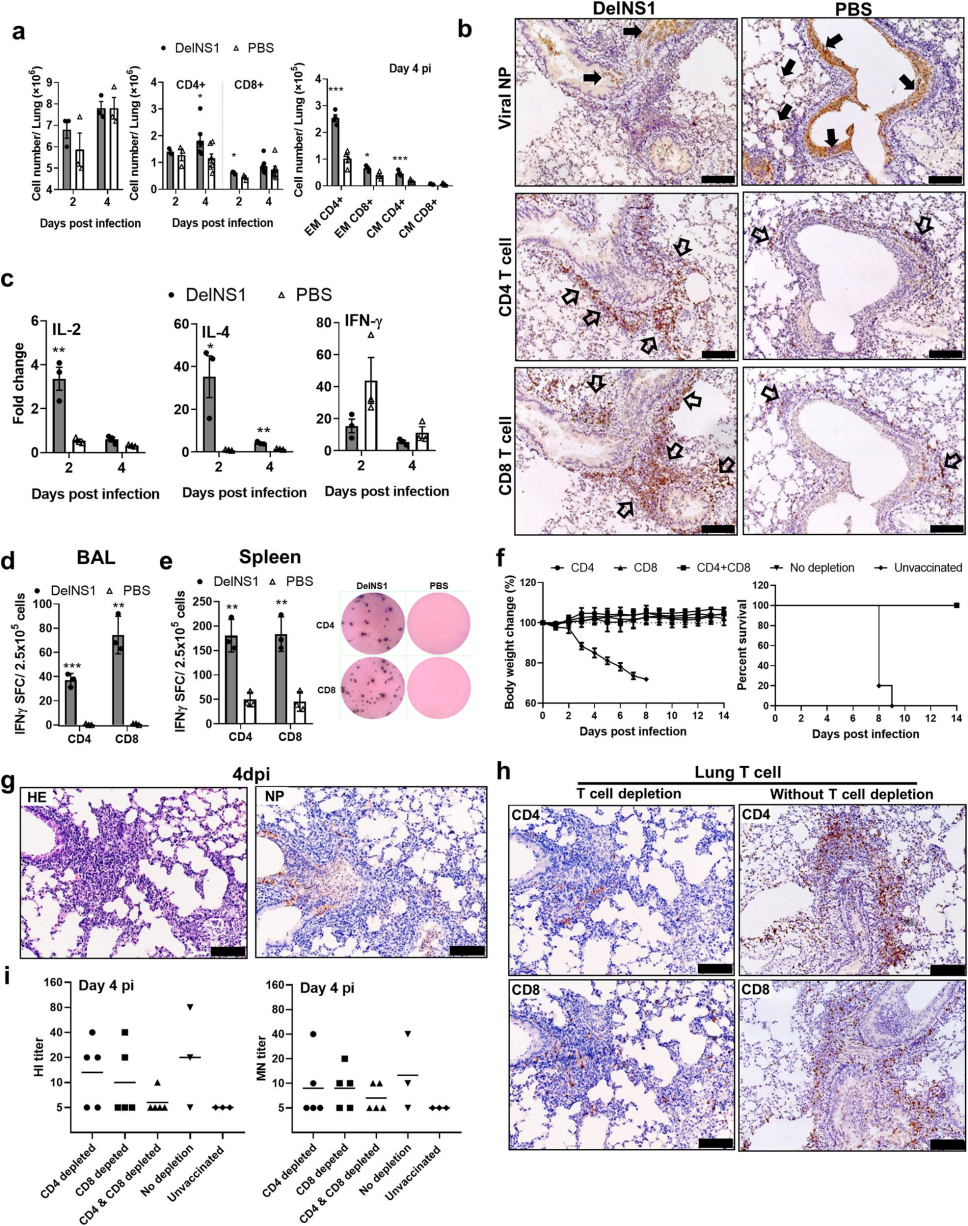
T cell responses in vaccinated mouse lungs. Mice were challenged with 10x LD_50_ of mouse-adapted A/Hong Kong/415742Md virus at 14 days after i.d. vaccination. Bronchoalveolar lavage fluid (BAL), lung, spleen, and serum samples were collected at 2 and 4 dpi. **a** T cell composition in mouse lung single-cell suspension by flow cytometry assays. Effector memory (EM) T cells were identified as CD3^+^, CD4^+^/CD8^+^, CD44^+^, and CD62L^−^ cells, whereas central memory (CM) T cells were identified as CD3^+^, CD4^+^/CD8^+^, CD44^+^, and CD62L^+^ cells. *n* = 3–5 mice. Error bars indicate standard error of mean. ^*^p < 0.05; ^**^p < 0.01; ^***^p < 0.001 when compared with PBS group by two-way ANOVA. **b** CD4^+^ and CD8^+^ T cell distribution in lung sections at 2 dpi. Representative images of immunohistochemically stained NP, CD4, and CD8. In vaccinated mouse lung, some viral NP-expressing cells were observed in the bronchiolar epithelium (solid arrows), extensive CD4^+^ and CD8^+^ T cell infiltration around the same bronchiole (open arrows). In PBS control mouse lung, abundant viral antigen-expressing cells in the entire epithelium layer (solid arrows) while CD4^+^ and CD8^+^ T cell infiltration were much less frequent (open arrows). Scale bars = 100 µm. **c** Relative expression levels of IL-2, IL-4, and IFN-γ in lung at 2 dpi by real-time RT-PCR comparing to uninfected lung samples. Viral-specific interferon-γ producing CD4^+^ and CD8^+^ T cells in (**d**) BAL and spleen (**e**) taken at 4 dpi. In vitro T cell epitope stimulation with CD4 epitope and CD8 T epitope from influenza NP (CD4 T epitope peptide: RLIQNSITIERMVLS; CD8 epitope peptide: TYQRTRALV) were performed for 48 h. Right-hand side is the representative images from EISPOT assay. *n* = 3 per group. Error bars indicate standard error of mean. ^*^p < 0.05; ^**^p < 0.01 when compared with PBS group by two-way ANOVA. **f** Antibody-mediated T cell depletion in vaccinated mice did not compromise vaccine-induced protection against A/Hong Kong/415742Md challenge, no weight loss (left) or lethality (right) were observed after virus challenge. **g** Representative images of H&E (left) and immunohistochemistry stained viral NP in the T cell-depleted mouse lung at 4 dpi. Scale bars = 100 µm. **h** Immunohistochemistry stained CD4, CD8 T cells, respectively, in the T cell-depleted or not depleted mouse lungs at 4 dpi. Scale bars = 100 µm. **i** Serum antibody titer, HI (left), MN (right) tested at 4 dpi in T cell-depleted or not depleted mice, and control mice. *n* = 3–5 per group.

Next, we studied the role of T cell responses in the protection of vaccinated mice after H1N1/415742Md challenge. We detected significantly more CD4^+^ and CD8^+^ T cells at 2 and 4 dpi in vaccinated mouse lungs by flow cytometry (Fig. 6a, middle), among which there were particularly higher proportion of memory CD4^+^ and memory CD8^+^ T cells (Fig. 6a, right). Immunohistochemistry staining showed intensive infiltration of CD4^+^ and CD8^+^ T cells around the infected airway structures in vaccinated mouse lung comparing to the unvaccinated controls at 2 dpi (Fig. 6b). Significantly higher expression levels of IL-2 and IL-4 in the lung at 2 dpi (Fig. 6c) also indicated the activation of T cell responses in the vaccinated mouse lung tissues. Further, using CD4 or CD8 T cell epitope from influenza virus nucleoprotein (CD4 T epitope peptide: RLIQNSITIERMVLS; CD8 epitope peptide: TYQRTRALV) stimulation^16,22^, we detected significantly higher number of interferon-γ producing virus-specific CD4 and CD8 T cells from vaccinated mouse bronchoalveolar lavage (BAL, Fig. 6d). Similarly, virus-specific T cells were significantly increased in the spleen of vaccinated mice (Fig. 6e).

To further understand the protective role of T cells, we performed T cells depletion in vaccinated mice by intraperitoneal administration of monoclonal antibodies against CD4 and/or CD8 before virus challenge (Supplementary Fig. 2a, b). We found that antibody-mediated T cell depletion did not compromise i.d. DelNS1-LAIV-induced protective effects (Fig. 6f). Mouse lung histology showed inflammatory infiltration and viral antigen expression (Fig. 6g), which were similar to those in vaccinated mice without T cell depletion (in Fig. 2c). However, the density of CD4^+^ and CD8^+^ T cells presented at the infected site was dramatically reduced after T cell depletion (Fig. 6h). These data suggested that there existed other protective mechanisms besides T cell-mediated responses, as we did detect HI and neutralizing antibody from mouse serum after T cell depletion (Fig. 6i).

### Intradermal vaccination of DelNS1-LAIV induced viral-specific B cell responses in challenged mice

**Fig. 7:**
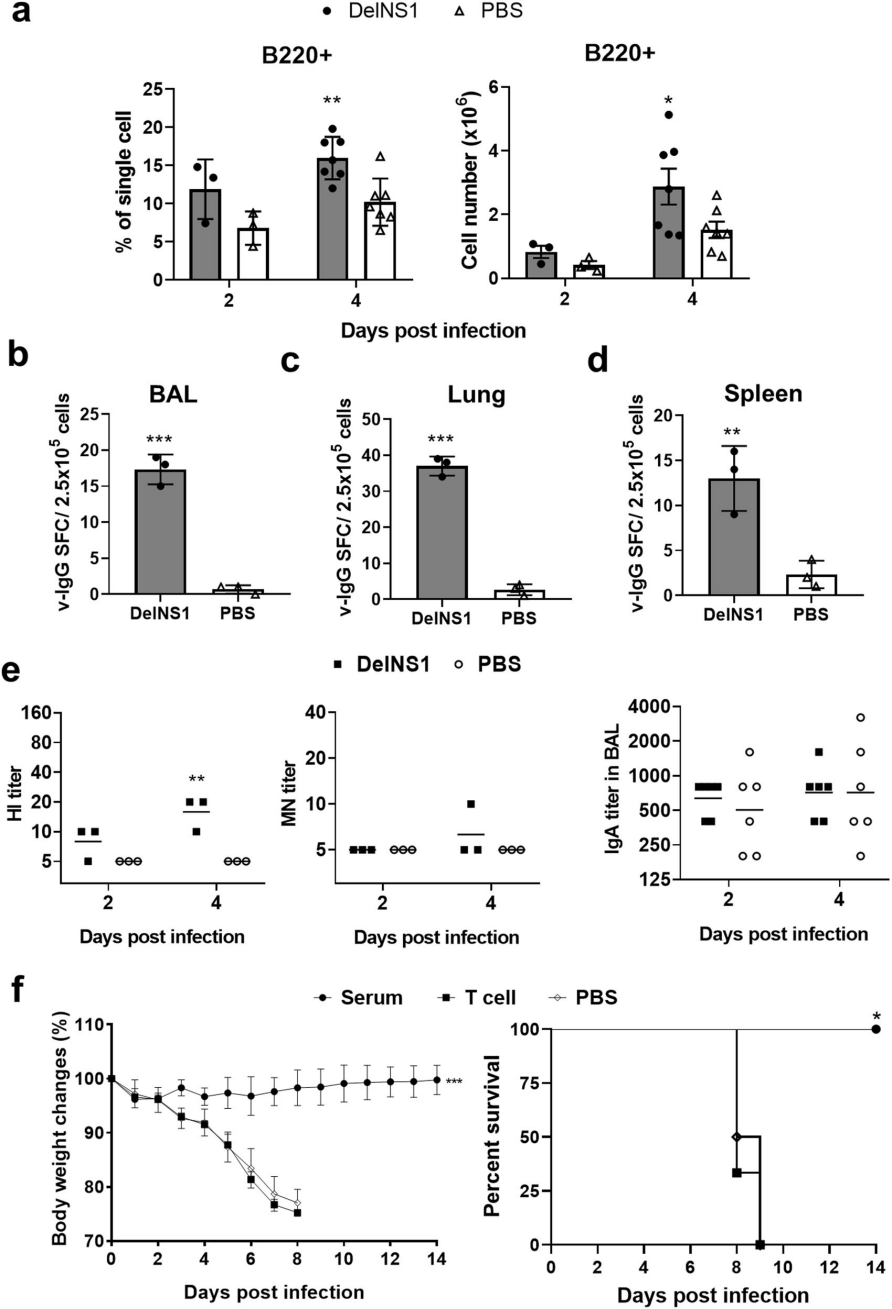
B cell responses in vaccinated mice and passive transfer of immunized serum and T cells for protection. Mice were challenged with 10x LD_50_ of mouse-adapted A/Hong Kong/415742Md virus at 14 days after i.d. vaccination. Bronchoalveolar lavage fluid (BAL), lung, spleen, and serum samples were collected at 2 and 4 dpi. **a** B cell composition in mouse lung single-cell suspensions determined by flow cytometry assays. *n* = 3 or 7 per group. ^*^p < 0.05; ^**^p < 0.01 when compared with PBS group by two-way ANOVA. ELISPOT assay detected virus-specific IgG producing B cells in the BAL (**b**), lung single-cell suspension (**c**), and spleen (**d**) after in vitro stimulation for 48 h by influenza antigen. *n* = 3 per group. Error bars indicate standard error of mean. **e** GMT of HI and MN antibody against A/Hong Kong/415742/2009 detected in vaccinated and control mouse sera at 2 and 4 dpi. IgA antibody in BAL samples was determined by ELSIA. *n* = 3 per group. ^*^p < 0.05; ^**^p < 0.01 when compared with PBS group by two-way ANOVA. **f** Passive transfer of pooled vaccinated mouse serum or spleen T cells to naive mice before A/Hong Kong/415742Md challenge (3x LD_50_). Serum or spleen T cells obtained from vaccinated mice 28 days after vaccination were pooled; 400 μl of serum (pooled from 9 vaccinated mice, HI titer = 1:80) or 1 × 10^7^ purified pan T cells were transferred to groups of naive mice via intraperitoneal injection before challenged with 3x LD_50_ of A/Hong Kong/415742Md virus. Body weight changes and survival were monitored for 14 days. Data presented are from two experiments. *n* = 6 per group. Error bars indicate standard deviation.

Flow cytometry analysis of lung single-cell preparations showed significantly more B cells in the vaccinated mouse lungs at 2 and 4 days after H1N1/415742Md challenge (Fig. 7a), which suggested fast B cell recruitment to the infected lungs. ELISPOT assay further demonstrated a significantly higher number of virus-specific IgG producing B cells in the BAL, lung, and spleen single-cell samples (Fig. 7b–d). In support of viral-specific B cell responses, at 4 dpi after H1N1/415742Md challenge, vaccinated mice had higher titers of serum HI and neutralizing antibody comparing to the control mice (Fig. 7e); while the mucosal IgA antibody response seemed less effective, since ELISA assay detected similar level of IgA in BAL of vaccinated and non-vaccinated control mice at day 2 and 4 dpi (Fig. 7e, right).

Overall, our data indicated that both humoral and cellular adaptive immune responses were elicited by i.d. DelNS1-LAIV vaccination. To further analyze the protective roles for the both arms of immunity in mice, we performed passive transfer of T cells or serum collected from vaccinated mice to naive mice before virus challenge. Our data showed total spleen T cells isolated 28 days after two doses vaccination could not protect the naive mice from lethal virus challenge (Fig. 7f) while transfer of pooled serum samples protected the naive mice against virus challenge (Fig. 7f).

## Discussion

The aim of this study is to investigate the efficacy and mechanism of i.d. vaccination of LAIV. We demonstrated that i.d. injection of DelNS1-LAIV induced potent protective responses in mice and conferred full protection not only against homologue virus challenge but also against heterologous viruses with protection lasting for at least 6 months. Our results showed that both B and T cell immunity were activated and the protection effects are mainly attributed to serum antibody responses. Our results broaden the potential application of LAIV to subjects who are not suitable for i.n. vaccination.

LAIV is mostly administered via intranasal spray to deliver vaccine virus to the upper respiratory tract which mimics the natural infection process and induces effective host immune responses^4^. Intradermal administration of live attenuated vaccines has only been used for BCG and some types of inactivated vaccines. For example, WHO recently recommends the intradermal route for post-exposure prophylaxis inactivated rabies, based on studies that i.d. administration offers an equally safe and efficacious alternative to intramuscular vaccination^23,24^. In addition, i.d. vaccination offers a dose-sparing benefit which reduces the volume of vaccine by 60–80% comparing with intramuscular vaccination^25^. The immunological basis for i.d. vaccination comes from the recognition of skin being an important part of the immune system^26,27^. Skin tissue contains various types of immune cells in the dermal layer, for example, dermal dendritic cells, Langerhans cells, resident T cells and B cells^26,28^, all of which play vital roles against natural infection, in capturing and presenting antigens to lymphoid organs during vaccination. Our DelNS1-LAIV lacked the NS1 gene and replicated well in MDCK cells at 33 °C^16^, which is the temperature of skin tissues in human and mouse^17,18^. We showed here that DelNS1-LAIV had limited replication in the skin tissue within 24 h after i.d. injection. DelNS1-LAIV showed no virus replication in skin-draining lymph nodes, which is certainly a safety benefit to avoid undesired side effects. The strong immune response in both skin tissues and skin-draining lymph nodes can be attributed to skin antigen-presenting cells, which mediated antigen transportation to the lymphoid tissues. In vitro infection of MoDCs by DelNS1-LAIV demonstrated an abortive feature of DelNS1-LAIV replication in dendritic cells at 33 °C while promoted strong MoDCs activation as shown by the high-level expressions of cytokine/chemokine after infection. Further, the limited replication of DelNS1-LAIV in skin tissue is crucial for the induction of subsequent adaptive immunity as the protective effects were lost when the vaccine virus was UV-inactivated.

As indicated by the body weight and survival rate after lethal-dose virus challenge, i.d. DelNS1-LAIV vaccination induced a similar level of protection as those vaccinated via the intranasal route. However, we found that i.d. vaccination-induced immunity is not sterilising in mice because virus titer was detected in mouse lungs after challenges. A possible explanation for this is that i.d. injection of live vaccine may induce lower mucosal antibody (IgA) comparing to i.n. vaccination of LAIV which were reported to have stronger mucosal immunity^3,4^. This could also partially be due to the earlier virus challenge experiment performed at 14 days after i.d. vaccination, while in our previously reported i.n. vaccination, virus challenge experiments were performed at 21 days after vaccination^16^. Nevertheless, i.d. vaccinated mice showed significantly reduced lung viral load and viral antigen NP was restricted to the bronchial epithelial layers. These findings indicated that the infection was well controlled after i.d. vaccination. Furthermore, lethal-dose virus challenge only caused mild degree of epithelial cell death and lung inflammatory cytokine responses. These findings indicated that the i.d. vaccination approach provided protection against severe disease upon infections.

Serum HI and neutralizing antibody titers are often used as standard criteria to evaluate influenza vaccine immunogenicity, especially when vaccination was given via injections. Intradermal vaccination of DelNS1-LAIV induced serum HI and MNT antibodies in mice. A single dose of i.d. vaccination induced low HI and neutralizing titers at 14 days after vaccination while the titers elevated significantly after the second dose. The HI or MN antibody titers were not particularly high in our model at 14 days after the second vaccination. However, passive transfer of immunized mouse serum to naive mice fully protected the naive mice against lethal virus challenge. Further analyses of the serum antibody showed virus-specific IgG, IgG2a, and IgG1, which indicated that both Th1 and Th2 antibody responses were elicited in the i.d. vaccinated mice. It is well known that comparison of results for serum HI and MNT antibody titers against influenza vaccination or infection between different studies are problematic due to the nature of these assays and different laboratory settings. Together with the high protective effects, evaluation of serum HI or MN titers alone may underestimate the effects of i.d. LAIV; similar inconsistency of HI titers with protection were also observed from LAIV human clinical studies^29^. Virus-specific IgA in nasal secretions has been shown to be important for intranasal live vaccine-induced antibody responses^30^. However, we only detected a modest increase of IgA in the BAL of challenged mice at 2 and 4 dpi, which is not significantly different from that of the unvaccinated mice. This may suggest that the i.d. vaccination is more effective in inducing circulating antibodies than mucosal antibodies. In line with this observation, we found a better-preserved distal lung structure in challenged mice because circulating antibodies are more protective to the lower respiratory cells. Similarly, human studies with intramuscular vaccination of inactivated influenza vaccines usually elicited circulating antibodies, but not mucosal immune responses^31,32^, which may share similar underlying mechanisms with i.d. vaccination.

LAIVs have been shown to induce strong T cell responses via intranasal administration^4,33^. We demonstrated here that i.d. vaccination with LAIV induced virus-specific CD4^+^ and CD8^+^ T cells in lungs. Flow cytometry analysis indicated that they were mainly CD4^+^ and CD8^+^ effector T cells, which produced interferon-γ upon influenza T cell epitope stimulation in vitro. However, depletion of circulating CD4 and CD8 T cells through antibody injection in vaccinated mice did not affect the protection effect against lethal virus challenge. CD4^+^ and CD8^+^ T cells staining showed they were largely depleted from the lung at day 4 after virus challenge, hinting that T cells are not essential for early protection against virus challenge. This may also explain the observation that transfer of immunized T cells to naive mice could not protect them from virus challenge. Of note, we still observed some CD4 and CD8 T cells in the mediastinal lymph node (Supplementary Fig. 2c) and spleen tissue (Supplementary Fig. 2d) after T cell depletion, this suggested that our depletion procedure did not completely deplete T cells in the lymphoid tissue^34^. Therefore, there may be some T cell recruitment from 4 dpi onward as the last depleting antibodies were administrated at 3 dpi.

In conclusion, our study demonstrated that i.d. administration of LAIV induces B cell and T cell responses and offers an equally safe and efficacious alternative to i.n. vaccination. Along with the constant efforts to improve influenza vaccine using different strategies, our study opened up a new option for LAIV. Further studies for its safety and efficacy in human are warranted.

## Methods

### LAIV, animal, and viruses

LAIV (DelNS1-LAIV) was generated from A/California/04/2009(H1N1) by deleting the NS1 coding region as we previously reported^16^. Six to eight weeks old female BALB/c mice were obtained from Centre for Comparative Medicine Research, the University of Hong Kong. A/Hong Kong/415742/2009 H1N1 mouse-adapted strain (H1N1/415742Md)^35^, A/Puerto Rico/8/1934 H1N1 (PR8), mouse-adapted A/Anhui/1/2013 H7N9 (A/Anhui/1/2013m)^36^, and A/VNM/1194/2004 H5N1^37^ were used for virus challenge experiments after vaccination. Six to eight weeks old female BALB/c mice obtained from Laboratory Animal Unit of the University of Hong Kong were housed in specific pathogen-free animal facility with 12 h light–dark cycle and free access to standard pellet food and water. Vaccination and virus challenging experiments were performed in biosafety level 2, or level 3 animal laboratories at the Department of Microbiology. The experimental procedures were approved by the Committee on the Use of Live Animals in Teaching and Research, the University of Hong Kong (CULATR # 5095-19).

### Vaccination and virus challenge of mice

Mice were randomly divided into groups, the fur on the lower back were shaved to expose the skin. Next, 10^6^ PFU of DelNS1-LAIV diluted in 100 µl of PBS were injected i.d. with insulin syringe, 30–40 µl per injection site. The mice were observed twice daily to identify skin tissue damages. The second dose of vaccination was given via i.d. at 14 days after the first dose. Blood, skin, and lymph node samples were taken at 14 and 28 days after first dose vaccine, and 14 days after second does as well, for the analysis of vaccine-induced immune responses. Virus challenging experiments were performed under ketamine (100 mg/kg) and xylazine (10 mg/kg) anesthesia and intranasal inoculation of 10x LD_50_ of H1N1/415742Md, PR8, A/Anhui/1/2013m (H7N9), or A/VNM/1194/2004 (H5N1) at 14 or 28 days, 3 or 6 months after vaccination. The body weight and survival were monitored for 14 days after virus challenge. The lung viral load, histological changes, and immune responses were studied at 2 and 4 dpi.

### Determination of lung viral load by plaque assay

The lungs were taken at 2 and 4 dpi, the left-side lobes were homogenized in 1 ml of cold minimum essential medium (MEM) supplemented with 1% penicillin and streptomycin. Homogenates were clarified by centrifuge at 9000*g* for 10 min at 4 °C, supernatant were made aliquots and stored at −80 °C until use. For plaque assay, 10-fold serial diluted homogenates were inoculated into MDCK cell monolayer in 12-well plates followed by incubation at 37 °C for 1 h. The inoculum was removed by PBS washing and the cells were then overlaid with MEM containing 2% low-melting-point agarose and 2 µg/ml of L-1-*p*-tosylamide-2-phenylethyl chloromethyl ketone (TPCK)-treated trypsin. The cells were fixed in 10% formalin solution and stained with 1% crystal violet after 72 h incubation. The number of plaques were counted and calculated as PFU/ml.

### Real-time RT-PCR for cytokine gene expression

Total RNA was extracted from 350 µl of clarified lung homogenates using MiniBEST Universal RNA extraction kit (Takara Bio Inc., Shiga, Japan). cDNA was synthesized from 1 µg of RNA with oligo-dT primer and PrimeScriptTM RT kit (Takara). Cytokine gene expression levels were determined by real-time RT-PCR performed on a LightCycler 96 system (Roche Applied Sciences, Indianapolis, USA) using gene-specific primers (Supplementary Table 1) and SYBR Premix Ex Taq II (Takara). The expression of house-keeping gene β-actin or GAPDH was quantified in parallel for RNA normalization. The relative expression of the target genes was calculated by the ΔΔ Ct method.

### Histological examination of mouse lung tissue

For histological analysis, the right-side of mouse lungs were fixed in 10% formalin/PBS, processed, and embedded in paraffin blocks. Then, 4 µm thick tissue sections were stained by hematoxylin and eosin (H&E), and were examined by a blinded pathologist without the knowledge of the samples. The severity of bronchiolar epithelial cell death was assessed by the number of bronchiole sections with dead cell debris in the lumens and bronchiolar epithelial cell necrosis, and rated by a semi-quantitative scoring method as described in Supplementary Table 2^38^. Representative images were captured with Olympus BX53 semi-motorized fluorescence microscope equipped with CellSens software.

### Immunohistochemistry staining of lung sections

Rehydrated paraffin-embedded lung sections were treated with Antigen Unmasking Solution according the manufacturer’s instruction (Vector Laboratories Inc. Burlingame, CA, USA) to unmask the antigen. After blocking with 1% bovine serum albumin, the sections were incubated with mouse anti-influenza NP, rabbit anti-mouse CD4, or rabbit anti-mouse CD8 primary antibody (all from Abcam) at 4 °C overnight^39^, followed by biotin-conjugated secondary antibody (Calbiochem, Darmstadt, Germany) for 30 min at room temperature. Streptavidin peroxidase complex reagent (Vector Laboratories, Burlingame, CA) was then incubated at room temperature for 30 min followed by color development with 3, 3′-diaminobenzidine (DAB, Vector Laboratories). The slides were mounted and examined under microscope. Representative images were captured with Olympus BX53 semi-motorized fluorescence microscope.

### Passive transfer of immunized serum or spleen T cells to naive mice before virus challenge

Serum and splenocytes were collected 28 day after first dose i.d. vaccination and pooled for passive transfer; 400 μl of pooled serum (HA = 1:80) were intraperitoneally injected to naive mice at 18 h prior to and immediately before virus challenge. Next, 1 × 10^7^ of T cells purified using pan-T cell isolation kit (Miltenyi Biotec, Germany) were intraperitoneally injected to different groups of naive mice at 18 h prior to virus challenge. The mice were challenged by 3x LD_50_ of H1N1/415742Md virus.

### Hemagglutination inhibition (HI) assay and microneutralization (MN) assay

Mouse serum samples were treated with receptor-destroying enzyme (RDE) (Denka Seiken, Japan) overnight at 37 °C and inactivated at 56 °C for 30 min. For HI assay, 2-fold serially diluted serum was incubated with 4 HA unit of influenza virus at room temperature for 1 h and then incubated with 50 µl of 0.5% turkey red blood cells (Lampire Biological Laboratories, PA, USA). The plates were read after 30 min of incubation. For MN assay, diluted serum was incubated with 100 TCID_50_ of the virus at room temperature for 1 h and added to MDCK cells. After absorption for 1 h, virus inoculum was removed and cells were incubated with MEM with 2 µg/ml TPCK-treated trypsin at 37 °C. Cytopathic effects were evaluated at 72 h after incubation^40^.

### Enzyme-linked immunosorbent assay (ELISA)

For detection of virus-specific antibodies in mouse serum, 96-well immunoplates (Nunc-Immuno Modules; Nunc A/S, Roskilde, Denmark) were coated with inactivated virus particles (2 µg/ml) in 0.05 M NaHCO_3_ (pH 9.6) and incubated overnight at 4 °C. After blocking with 1% bovine serum albumin at 37 °C for 1 h, 2-fold serially diluted serum was added and incubated at 37 °C for 1 h. The plate was then washed 6 times with PBS containing 0.05% Tween-20, and incubated with horseradish peroxidase (HRP)-conjugated secondary antibodies (Life Technology, CA, USA) at 37 °C for 1 h. After color development with 3,3′,5,5′-tetramethylbenzidine solution (Life Technology, Carlsbad, CA, USA) for 15 min at 37 °C, the reaction was stopped with H_2_SO_4_. The optical density (OD) was read at 450 nm. The cut-off OD was set at the mean OD of uninfected serum at all dilutions plus 3 standard deviations. The highest sample dilution which produces an OD above this cut-off OD was taken as the antibody titer^41,42^.

### Flow cytometry assay

To determine the composition and activation of immune cells in the lymph nodes from vaccinated mice and lungs from infected mice, single-cell suspension were prepared and stained with fluorochrome-conjugated antibodies before fixing in 4% paraformaldehyde for flow cytometry assay. Single cells from lymph nodes were stained with CD45-PerCP/Cy5.5, B220-Brilliant Violet 421, CD11c-PE/Cy7, CCR7-APC, CD86-PE, and MHC-II (I-A/I-E)-Pacific blue antibodies (all from Biolegend) for the identification the activation of dendritic and B cells. Single cells from lung tissues were stained with CD45-APC/Cy7, B220-PerCP/Cy5.5, CD3-PerCP/Cy5.5, CD4-PE/Cy7, CD8-PE, CD44-APC/Cy7, and CD62L-Brilliant Violet 421 antibodies. Effector memory T cells were identified as CD3^+^, CD4^+^/CD8^+^, CD44^+^, and CD62L^−^ cells, whereas central memory T cells were identified as CD3^+^, CD4^+^/CD8^+^, CD44^+^, and CD62L^+^ cells. Stained cells were analyzed on LSRFortessa Cell Analyzer (BD Bioscience), data were analyzed using FlowJo software (TreeStar, Inc.).

### Enzyme-linked immunospot (ELISPOT) assay

To detect virus-specific IgG producing B cells, BAL, lung, and spleen were taken from vaccinated or non-vaccinated mice at day 4 post virus challenge. Single-cell suspensions from each sample were seeded into ELISPOT plates at 2.5 × 10^5^ cells/well, and incubated for 48 h with 100 μg of purified inactivated H1N1/415742Md virus (5 μg/ml) as antigen. IgG producing cells were then detected by alkaline phosphatase (AP)-conjugated goat anti-mouse IgG antibody. To determine virus-specific T cells, 2.5 × 10^5^ cells of single cells were seeded and stimulated with 3 μg/ml of H1N1 nucleoprotein CD4 epitope peptide (RLIQNSITIERMVLS) or CD8 epitope peptide (TYQRTRALV) for 48 h^16,22^, interferon-γ producing T cells were detected using mouse IFN-γ ELISpot BASIC kit (Mabtech, Inc., Stockholm, Sweden) following the manufacturer’s instructions.

### DelNS1-LAIV infection and activation of monocyte-derived dendritic cells (MoDCs)

MoDCs were generated from freshly isolated human CD14^+^ monocytes and cultured in RPMI1640 complete medium supplemented with IL-4 (10 ng/ml) and GM-CSF (10 ng/ml) for 6 days^19^. The cells were infected with DelNS1-LAIV and wild-type A/Hong Kong/415742/2009 virus at MOI of 2. Cell lysates were collected at 6 or 24 hpi for viral load and cytokine/chemokine gene expression analysis. Protocol for using buffy coats blood from healthy blood donors was approved by the Institutional Review Board of the University of Hong Kong (ref. no. IRB UW16-106).

### Statistical analysis

All statistical analyses were computed using Prism 8.0. Analysis between groups was performed by Student’s *t*-test or two-way ANOVA. Mouse survival rates were compared using the Kaplan-Meier method and the log-rank test. *p* Values of <0.05 were considered to be statistically significant.

### Reporting summary

Further information on research design is available in the Nature Research Reporting Summary linked to this article.

## Supplementary information

Supplementary information.

Reporting summary.

## Data Availability

The authors declare that the data supporting the findings of this study are available within the main and supplementary figures. All data are available from the corresponding author upon reasonable request.
