# Supplementary material for: Intradermal vaccination of live attenuated influenza vaccine protects mice against homologous and heterologous influenza challenges
Source: NPJ Vaccines. 2021 Aug 4;6:95. doi: 10.1038/s41541-021-00359-8 (PMC8339132; doi:10.1038/s41541-021-00359-8)
Supplement: Supplementary file 1 — Supplementary information. [file 41541_2021_359_MOESM1_ESM.pdf]

Supplementary Table 1. Sequences for primers used in real time RT-PCR assay

Target	Gene name	Forward primer (5' to 3')	Reverse Primer (5' to 3')
Influenza	*M gene*	CTTCTAACCGAGGTCGAAACG	GGCATTTTGGACAAAKCGTCTA
Human	*GAPDH*	ATTCCACCCATGGCAAATTC	CGCTCCTGGAAGATGGTGAT
Human	*IFN-α*	AGAATCACTCTCTATCTGAAAGAGAAGAAATA	TCATGATTTCTGCTCTGACAACCT
Human	*IFN-β*	AGTAGGCGACACTGTTCGTG	GCCTCCCATTCAATTGCCAC
Human	*IFN-γ*	CTAATTATTCGGTAACTGACTTGA	ACAGTTCAGCCATCACTTGGA
Human	*IL-6*	GGCTGCAGGACATGACAACT	ATCTGAGGTGCCCATGCTAC
Human	*TNF-α*	CAAGGACAGCAGAGGACCAG	TGGCGTCTGAGGGTTGTTTT
Human	*MIP-1α*	CATTCCGTCACCTGCTCAGAA	GGCTGCTCGTCTCAAAGTAGT
Human	*MIP-1β*	GTCTGTGCTGATCCCAGTGA	GCGGAGAGGAGTCCTGAGTA
Human	*RANTES*	AAGGAAGTCAGCATGCCTCT	TAAGCTCCTGTGAGGGGTTG
Human	*CD40*	CAAGCAGATTGCTACAGGGG	GCACAACCAGGTCTTTGGTC
Human	*CD80*	TCCACGTGACCAAGGAAGTG	CTCGTATGTGCCCTCGTCAG
Human	*CD86*	TGGGAATGCTGCTGTGCTTA	TTCAGAGGAGCAGCACCAGA
Mouse	*Β-actin*	ACGGCCAGGTCATCACTATTG	CAAGAAGGAAGGCTGGAAAAG
Mouse	*IL-1β*	GCCTTGGGCCTCAAAGGAAAGAATC	GGAAGACACAGATTCCATGGTGAAG
Mouse	*IL-6*	TGGAGTCACAGAAGGAGTGGCTAAG	TCTGACCACAGTGAGGAATGTCCAC
Mouse	*IL-2*	TTCAATTGGAAGATGCTGAGA	ATCATCGAATTGGCACTCAA
Mouse	*IL-4*	TTTTGAACGAGGTCACAGGA	AGCCCTACAGACGAGCTCAC
Mouse	*TNF-α*	ATAGCTCCCAGAAAAGCAAGC	CACCCCGAAGTTCAGTAGACA
Mouse	*IFN-α*	ARSYTGTSTGATGCARCAGGT	GGWACACAGTGATCCTGTGG
Mouse	*IFN-β*	TGGGAGATGTCCTCAACTGC	CCTGCAACCACCACTCATTC
Mouse	*IFN-γ*	AAGCGTCATTGAATCACACC	CGAATCAGCAGCGACTCCTT
Mouse	*MIP-1α*	CCCAGCCAGGTGTCATTTTCC	GCATTCAGTTCCAGGTCAGTG
Mouse	*MIP-1β*	TCTGTGCAAACCTAACCCCG	GAGAAACAGCAGGAAGTGGGA
Mouse	*MCP-1*	GGCTCAGCCAGATGCAGTTAA	CCTACTCATTGGGATCATCTTGCT
Mouse	*RANTES*	GAGGCTCTCTGCTGTCCATC	GCGGTTCCTTCGAGTGACAA
Mouse	*IP-10*	ATGACGGGCCAGTGAGAATG	GAGGCTCTCTGCTGTCCATC
Mouse	*CCL19*	CACTTGGCTCCTGAACCCC	AGTGAGTGAGCCTGAGAGACT
Mouse	*CCL21*	CGGCTGTCCATCTCACCTAC	CTCCATCACTGCCTTGGGTC
Mouse	*CXCL13*	CTCTCCAGGCCACGGTATTC	TTGGCACGAGGATTCACACA

Supplementary Table 2. Histopathological scoring criteria [22]

Histological score	Airway and alveolar cell necrosis	Cell infiltration
**Score 0**	Normal lung	Normal lung
**Score 1**	Airway epithelial cells necrosis limited in one lobe	Infiltration cells only seen in peribronchiolar area or alveolar wall in one lobe
**Score 2**	Airway epithelial cell necrosis in more than one lung lobes, with luminal cell debris	A few immune cells (1-5 cells) in alveolar space which located in focal area of one lobe
**Score 3**	Airway epithelial cell necrosis in more than one lung lobes; and with small area of alveolar wall collapse	More immune cells infiltration in alveolar space which can be seen in more than one lobe
**Score 4**	Airway epithelial cell necrosis in more than one lung lobes, and alveolar wall collapse in more than one lobes	Severe diffuse immune cell infiltration in alveolar space.

Supplementary Figure

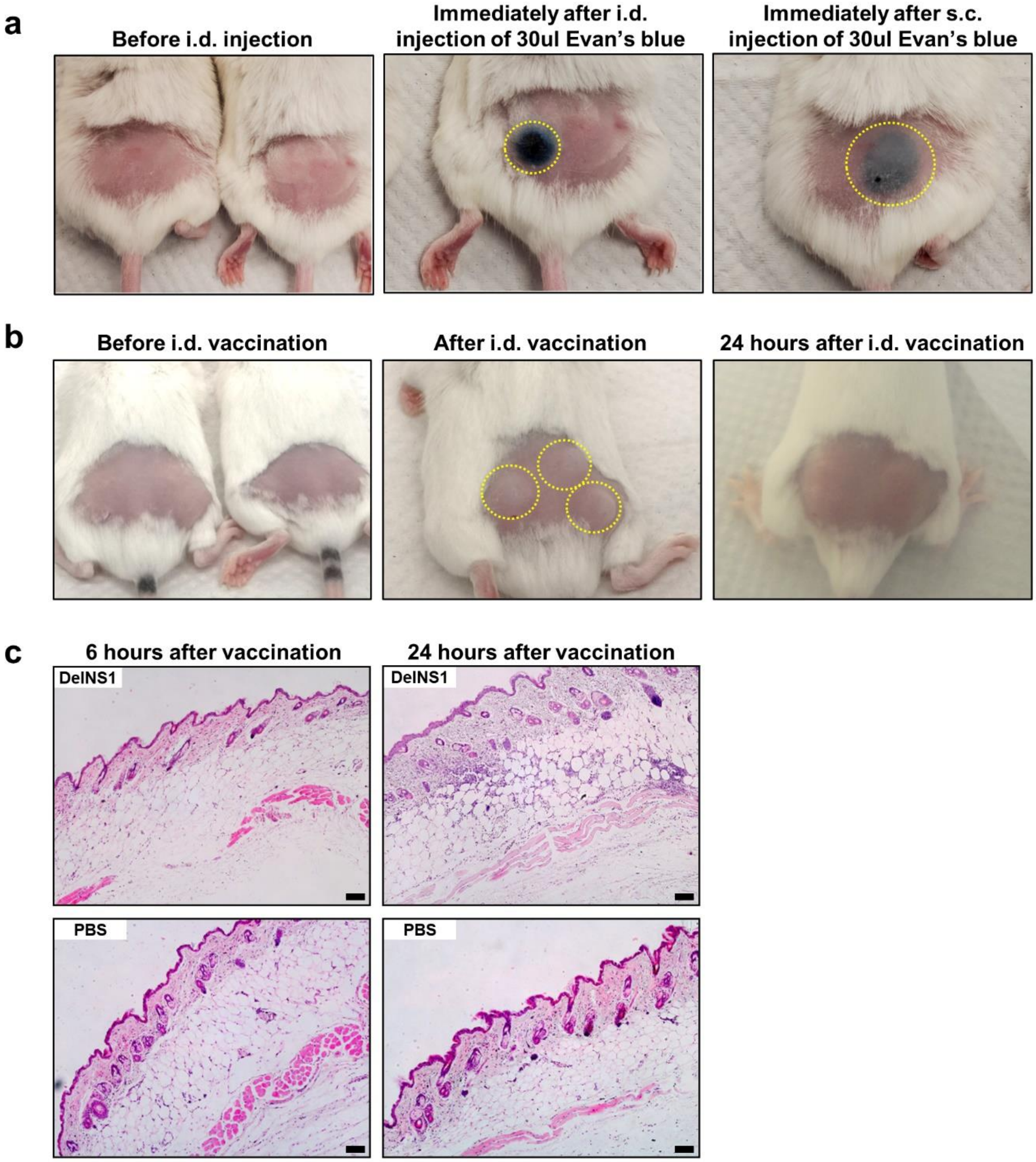

Supplementary Fig. 1. No gross and histological skin damages in mice after intradermal injection of 10^6 PFU DelNS1-LAIV. Fur on the lower back of the mice was shaved before injection to expose the skin. (a) Intradermal injection of Evan's blue dye (25mg/ml) to demonstrate intradermal distribution of injected material. Images of skin before injection (left), immediately after i.d. injection of 30ul of Evan's blue (middle) which showed a condensed blue dot with sharp edge, and immediately after subcutaneous injection (s.c.) of

30ul of Evan's blue (right) which showed a larger, greyish patch with blurred edge. (b)DelNS1-LAIV were injected to 3 sites in total volume of 100µL. Representative images of skin photos taken at different time showing the skin tissues of the injection site before, immediately after, and 24 hours after i.d. vaccination. (c) Skin tissue samples fixed in 10% formalin at 6 hours and 24 hours after i.d. vaccination. Representative images of haematoxylin and eosin (H&E) stained tissue sections showed no inflammatory damages. Scale bars = 100µm.

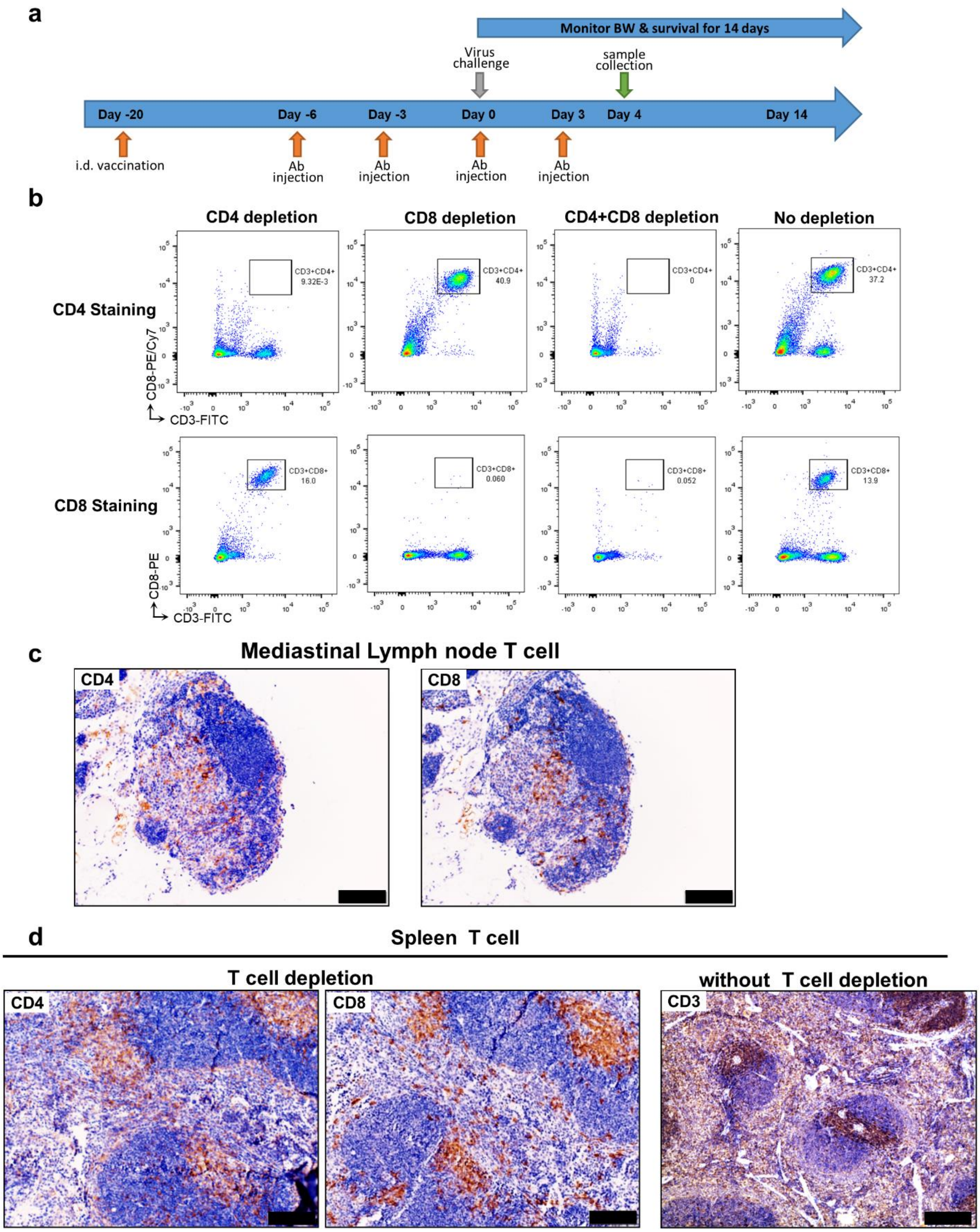

Supplementary Fig. 2. Depletion of CD4, CD8 T cells from vaccinated mice before virus challenge. (a) Illustration of the experimental protocol. Mice were intradermally injected with 10^6 PFU DelNS1-LAIV at day -20. 100μg of anti-mouse CD4, anti-mouse CD8β, or both CD4 and CD8β antibodies were injected

intraperitoneally at day -6 (14 days after vaccination), day -3, day 0, and day 3. Mice were challenged with 10 LD_{50} of H1N1/415742Md at day 0. (b) Representative dot plot of CD4+ and CD8+ T cells staining by flow cytometry at day 0 showing the $CD4^+$ and $CD8^+$ T cells were depleted in peripheral blood. (c) Images of immunohistochemistry stained CD4 and CD8 T cells in mediastinal lymph node from T cell depleted mice. Scale bar = 100μm. (d) Images of immunohistochemistry stained CD4 and CD8 T cells in spleen of T cell depleted mice. Scale bar = 100μm.
