# Supplementary material for: Intradermal vaccination of live attenuated influenza vaccine protects mice against homologous and heterologous influenza challenges
Source: NPJ Vaccines. 2021 Aug 4;6:95. doi: 10.1038/s41541-021-00359-8 (PMC8339132; doi:10.1038/s41541-021-00359-8)
Supplement: Supplementary file 2 — Reporting summary. [file 41541_2021_359_MOESM2_ESM.pdf]

nature research

Corresponding author(s): DBPR NPJVACCINES-01093R3

Last updated by author(s): Jun 19, 2021

Reporting Summary

Nature Research wishes to improve the reproducibility of the work that we publish. This form provides structure for consistency and transparency in reporting. For further information on Nature Research policies, see our Editorial Policies and the Editorial Policy Checklist.

Statistics

For all statistical analyses, confirm that the following items are present in the figure legend, table legend, main text, or Methods section.

n/a	Confirmed	
☐	☒	The exact sample size (n) for each experimental group/condition, given as a discrete number and unit of measurement
☐	☒	A statement on whether measurements were taken from distinct samples or whether the same sample was measured repeatedly
☐	☒	The statistical test(s) used AND whether they are one- or two-sided<br>*Only common tests should be described solely by name; describe more complex techniques in the Methods section.*
☐	☒	A description of all covariates tested
☐	☒	A description of any assumptions or corrections, such as tests of normality and adjustment for multiple comparisons
☐	☒	A full description of the statistical parameters including central tendency (e.g. means) or other basic estimates (e.g. regression coefficient) AND variation (e.g. standard deviation) or associated estimates of uncertainty (e.g. confidence intervals)
☐	☒	For null hypothesis testing, the test statistic (e.g. F, t, r) with confidence intervals, effect sizes, degrees of freedom and P value noted<br>*Give P values as exact values whenever suitable.*
☒	☐	For Bayesian analysis, information on the choice of priors and Markov chain Monte Carlo settings
☒	☐	For hierarchical and complex designs, identification of the appropriate level for tests and full reporting of outcomes
☒	☐	Estimates of effect sizes (e.g. Cohen's d, Pearson's r), indicating how they were calculated

Our web collection on statistics for biologists contains articles on many of the points above.

Software and code

Policy information about availability of computer code

Data collection	Olympus CellSens<br>BD FACSDiva<br>LightCycler® 96 System
Data analysis	Prism - GraphPad Software Inc.<br>Flowjo - TreeStar, Inc

For manuscripts utilizing custom algorithms or software that are central to the research but not yet described in published literature, software must be made available to editors and reviewers. We strongly encourage code deposition in a community repository (e.g. GitHub). See the Nature Research guidelines for submitting code & software for further information.

Data

Policy information about availability of data

All manuscripts must include a data availability statement. This statement should provide the following information, where applicable:

- Accession codes, unique identifiers, or web links for publicly available datasets
- A list of figures that have associated raw data
- A description of any restrictions on data availability

The authors declare that the data supporting the findings of this study are available within the main and supplemental figures. All data are available from the corresponding author upon reasonable request.

Field-specific reporting

Please select the one below that is the best fit for your research. If you are not sure, read the appropriate sections before making your selection.

☒ Life sciences ☐ Behavioural & social sciences ☐ Ecological, evolutionary & environmental sciences

For a reference copy of the document with all sections, see nature.com/documents/nr-reporting-summary-flat.pdf

Life sciences study design

All studies must disclose on these points even when the disclosure is negative.

Sample size	The sample size (n) of each experiment is provided in the corresponding figure legends in the main manuscript and supplementary materials. We have good reproducibility between replicates and sample sizes were chosen to support meaningful conclusions.
Data exclusions	No data was excluded from the analyses.
Replication	For in vitro experiments, all were performed for 2-3 times. For in vivo experiments, each experiment presented in the paper was performed at least twice, each time having 3-7 animals per group.
Randomization	Animals were assigned randomly into experimental and control groups.
Blinding	The investigators were not blinded. The pathologists for histological examination, and operator for ELISPOT analysis were blinded from grouping information.

Reporting for specific materials, systems and methods

We require information from authors about some types of materials, experimental systems and methods used in many studies. Here, indicate whether each material, system or method listed is relevant to your study. If you are not sure if a list item applies to your research, read the appropriate section before selecting a response.

Materials & experimental systems

n/a	Involved in the study	
☐	☒	Antibodies
☒	☐	Eukaryotic cell lines
☒	☐	Palaeontology and archaeology
☐	☒	Animals and other organisms
☐	☒	Human research participants
☒	☐	Clinical data
☒	☐	Dual use research of concern

Methods

n/a	Involved in the study	
☒	☐	ChIP-seq
☐	☒	Flow cytometry
☒	☐	MRI-based neuroimaging

Antibodies

Antibodies used

Mouse anti-influenza NP (in-house)

Rat anti-CD45-PerCP-Cy5.5, Biolegend, Cat: 103132, Clone: 30-F11
Rat anti-CD45-APC/Cy7, Biolegend, Cat: 103116, Clone: 30-F11
Hamster anti-CD11c-PE/Cy7, Biolegend, Cat: 117318, Clone: N418
Rat anti-CCR7-APC, Biolegend, Cat: 120108, Clone: 4B12
Rat anti-CD86-PE, Biolegend, Cat: 105008, Clone: GL-1
Rat anti-I-A/I-E-Pacific Blue, Biolegend, Cat: 107620, Clone: M5/114.15.2
Rat anti-B220-PerCP/Cy5.5, Biolegend, Cat: 103233, Clone: RA3-6B2
Hamster anti-CD3-PerCP/Cy5.5, Biolegend, Cat:100328, Clone: 145-2C11
Rat anti-CD8-PE, Biolegend, Cat: 100422, Clone: 53-6.7
Rat anti-CD44-APC/Cy7, Biolegend, Cat: 103028, Clone: IM7
Rat anti-CD62L-BV421, Biolegend, Cat: 104436, Clone: MEL-14

Rabbit anti-CD4, abcam, Cat: ab183685, Clone: MT310
Rabbit anti-CD8 alpha, abcam, Cat: ab209775, Clone: ERP20305

Validation

Mouse anti-influenza NP (in-house) was validated by IHC/IF staining of influenza-infected and mock-infected MDCK cells and mouse lung tissues. This antibody has been used in many publications (Zhang et al. 2020 CID https://doi.org/10.1093/cid/ciaa1747; Lee et al. 2019 CDDis https://www.nature.com/articles/s41419-019-1684-0; Lee et al. 2015 CVI https://cvi.asm.org/content/22/12/1235.short; Li et al. 2014 Plos One https://doi.org/10.1371/journal.pone.0107966)

All antibodies from Biolegend applied to flow cytometry staining were validated by manufacturer by staining of mouse stimulated or unstimulated splenocytes with isotype control.

All antibodies from abcam were validated by manufacturer for IHC-P application in mouse.

Animals and other organisms

Policy information about studies involving animals; ARRIVE guidelines recommended for reporting animal research

Laboratory animals	6-8 weeks old female Balb/c mice
Wild animals	The study did not involve wild animals.
Field-collected samples	The study did not involve samples collected from the field.
Ethics oversight	Animal protocols were reviewed and approved by the Committee on the Use of Live Animals in Teaching and Research, the University of Hong Kong (CULATR # 5095-19).

Note that full information on the approval of the study protocol must also be provided in the manuscript.

Human research participants

Policy information about studies involving human research participants

Population characteristics	Health Blood Donor from Hong Kong Red Cross
Recruitment	Not apply to this study
Ethics oversight	Ethical approval obtained from Institutional Review Board of the University of Hong Kong (ref no. IRB UW16-106)

Note that full information on the approval of the study protocol must also be provided in the manuscript.

Flow Cytometry

Plots

Confirm that:

- [x] The axis labels state the marker and fluorochrome used (e.g. CD4-FITC).
- [x] The axis scales are clearly visible. Include numbers along axes only for bottom left plot of group (a 'group' is an analysis of identical markers).
- [x] All plots are contour plots with outliers or pseudocolor plots.
- [x] A numerical value for number of cells or percentage (with statistics) is provided.

Methodology

Sample preparation	Lung single cell suspensions were isolated according to the instruction of mouse lung dissociation kit using a gentleMACS Dissociator (Miltenyi Biotec). For lymph nodes and spleens, tissues were disrupted by gentle mechanical disruption using the plunger of a 3-ml spring and passed through 70µm cell strainer to obtained single cells suspension. Red blood cells were lysed with RBC lysis buffer and washed with PBS.
Instrument	BD LSR Fortessa cell analyser
Software	FACSDiva (BD Bioscience) was used to collect the flow cytometry raw data. Flowjo (TreeStar, Inc)was used to analyze the flow cytometry raw data.
Cell population abundance	Sorting of the cells or enrichment of one single cell type were not performed in this study.
Gating strategy	Cell population of interest was identified and cells debris were excluded by forward scatter area versus side scatter area (FSC-A vs SSC-A) gating. Doublets were then excluded by forward scatter height (FSC-H) versus FSC-A and side scatter height (SSC-H) versus SSC-A gating. The singlet cell expressing specific markers were identified by fluorochrome-conjugated antibody listed in the supplementary materials. Isotype control antibodies were used to define the marker-negative population.

- [x] Tick this box to confirm that a figure exemplifying the gating strategy is provided in the Supplementary Information.
